# Panel Pay and Extended Services

**Published:** 1923-07

**Authors:** R. W. Harris


### PANEL PAY AND EXTENDED SERVICES.—III.

By R. W. HARRIS.

IF we ignore the scrap-the-lot critic of the panel system—who might be worthy of attention if he had any constructive suggestions to put forward—and turn to the criticism in more responsible quarters, one definite point is beginning to emerge. There is a demand for a fuller medical service for the insured. It comes alike from those who claim to speak for the insured and from the doctors themselves.

### An Important Distinction.

It appears to me to be very important that the distinction between a fuller service and a better service within the existing range should be closely observed, in considering the question of the panel doctor's pay. That there is a broad relation between the pay and the quality of the service is not to be denied, and the depression of the pay below a fair and just level would obviously cause good men to drop out, and would mean a disgruntled service from those who would remain. But you cannot, by the payment of an extra sixpence or two extra sixpences, purchase an improvement in human nature. It will not effect any change in the spots of the leopard or in the skin of the Ethiopian. To say, therefore, that $x$ is fair and just pay for what you are getting now, but you will gladly pay $x + y$ for a better service, is rather meaningless. And to say that you will pay $x + y$ for a fuller service is to confuse the question of panel pay with that of payment for extended services which would not, in general, affect the pockets of the panel doctors.

### The Two Voices.

The "National Insurance Gazette," in its issue of June 9th, says: "The general level of the service does not, in our opinion, justify a fee of 9s. 6d. The correct and fair amount is, in our opinion, 7s. 6d., but we would gladly pay more if the service were substantially improved. . . . To justify 9s. 6d. we must have extensions and improvements worth 2s." The reports in the newspapers of the same date state that the Conference of Panel Committees passed a resolution: "That any rate of remuneration lower than that given as a result of the 1920 arbitration (11s.) was less than adequate for the service rendered. Also, that at the earliest time, with due regard to financial considerations, there should be an extension of the medical service to matters beyond general practitioner range."

### A Burden on Industry.

I express no opinion as to the merits of the respective views that 7s. 6d. is the appropriate fee or that anything less than 11s, is inadequate; it would perhaps be improper for me to do so in view of my past associations. At the moment, 9s. 6d. is paid and Sir Alfred Mond is reported recently to have said that it has always puzzled him to know how this amount (which he settled with the doctors) was arrived at. Certain facts may, however, be recited: That 11s. was determined by arbitration in the early part of 1920; that the cost of living has since fallen steadily; that the reduction of 1s. 6d. was accepted by the doctors at a time when the Geddes axe was cutting pretty big chips off everywhere else; that there are no very encouraging signs of economic recovery; and that, however healthy the condition of insurance funds, insurance contributions are a burden on industry.

### A Possible Basis of Settlement.

I have purposely not looked up the index figures of the cost of living to see how the 11s. would scale down, at the present time, if adjusted by reference to those figures; partly, because the cost of living is only one factor in the calculation, and also because I prefer to use amounts for purposes of illustration only. The doctors, quite reasonably, ask for a five-years' settlement, and, as well as the insured, they need a fuller service. The provision of X-ray and laboratory facilities, the obtaining of second opinions, and some other consultant and specialist services, would afford that fuller service. Some of these extra services, for the lack of which the panel service gets into so much bad odour, would be within the competence of some doctors, specially skilled, who are already panel practitioners, but generally it would be necessary to obtain them outside their ranks. That involves paying for them separately on an agreed scale of fees to all doctors accepting such a scale and registering accordingly for the purpose. The panel fee must remain the general flat-rate fee for services within the competence of general practitioners. For services outside the competence of practitioners generally—although within the competence of some—there must be separate payment, separate organisation, properly co-ordinated with the panel system, and some, but one would hope very few, conditions embodied in regulations.

### Specialist Services.

Suppose, then, for purposes of illustration, that 11s., adjusted to meet the change in general conditions since 1920, becomes 9s. 6d. If the doctors were to agree to a reduction of 1s. from the 9s. 6d. on the understanding that a further 1s. were found from insurance funds, to create a pool at 2s. a head of the insured population, equivalent to £1,500,000, a reduction of 6d. would produce, with a new 1s., a sum of about £1,100,000. Either amount would enable very substantial contributions to be made towards the cost of the extra services to which I have referred; and those panel doctors recognised as possessing special skill and as able, consistently with the best interests of the patient, to render specialist services, would participate in the separate fund together with other specialists. I have in mind, at the outset, nothing more than an agreed scale of fees and a contribution, *pro rata*, so far as the fund would allow, the arrangements being improved and extended in the light of experience and of the further assistance which insurance funds could afford. In a position of admitted difficulty, I put forward this suggestion with diffidence, as at any rate worthy of careful consideration.

